# Life’s Crucial 9 and psoriasis: a mediation analysis of systemic inflammation response index using NHANES cohort data

**DOI:** 10.3389/fimmu.2025.1524382

**Published:** 2025-04-15

**Authors:** Xinyan Liu, Qirui Deng, Hongfeng Tang

**Affiliations:** Department of Dermatology, Shunde Hospital, Southern Medical University (The First People’s Hospital of Shunde), Foshan, Guangdong, China

**Keywords:** LC9, SIRI, psoriasis, NHANES, mediation analysis

## Abstract

**Background:**

Psoriasis is a chronic, recurrent, inflammatory disease. The aim of this study was to investigate the association between the Life’s Crucial 9 (LC9) score and the risk of psoriasis and to examine the mediating role of system inflammation response index (SIRI).

**Methods:**

Utilizing data from the National Health and Nutrition Examination Survey (NHANES) spanning 2009 to 2014, this study employed multifactor logistic regression, restricted triple spline analysis, and various subgroup analyses to evaluate the correlation between LC9 scores and the occurrence of psoriasis. Furthermore, mediation analyses were executed to investigate SIRI’s possible intermediary effect in the linkage between LC9 scores and psoriasis.

**Results:**

This study included 11,499 US adults with 345 psoriasis patients. In fully adjusted models, a notable inverse relationship was identified between the LC9 score and the prevalence of psoriasis (OR = 0.90, 95% CI: 0.80, 0.99). After adjusting for all covariates, no significant interactions were found in the subgroup analyses. In addition, mediation analysis showed that SIRI mediated the association between LC9 and psoriasis (mediation ratio: 7.02%, p = 0.004).

**Conclusions:**

Higher LC9 scores reduce the risk of psoriasis. And, this association was mediated by SIRI. This suggests that we should actively regulate our lifestyles to improve cardiovascular health, thereby preventing and delaying the onset of psoriasis.

## 1 Introduction

Psoriasis ranks among the prevalent chronic dermatological inflammatory disorder with a complex pathogenesis that affects approximately 3% of adults in the United States (1, 2). In addition, recent studies have shown that psoriasis, as a chronic, relapsing, systemic immunoinflammatory disease, is an important cause of reduced quality of life, disability, and contributes to psychological problems (3, 4). Studies have shown that psoriasis places a heavy economic and disease burden on global public health (5–7). Therefore, modulating early controllable risk factors is extremely critical to reduce the incidence of psoriasis and mitigate the risk of its comorbidities.

Introduced in 2010 by the American Heart Association, the Life’s Simple 7 (LS7) scale serves as a tool to evaluate cardiovascular health (8). It was subsequently proposed in 2022 to incorporate a new factor, sleep, called Life’s Essential 8 (LE8). Recent research has independently linked mental health to both the risk and outcomes of cardiovascular disease (CVD), highlighting a previously overlooked critical factor (9). Consequently, the introduction of a new evaluation tool, LC9, now integrates a mental health perspective with existing measures. Research by Lu et al. demonstrates an association between LC9 scores and all-cause mortality from cardiovascular conditions (10). However, the association between LC9 and psoriasis is still unknown.

Earlier research has established a significant positive correlation between the SIRI and psoriasis risk (OR = 1.48,95% CI: 1.12, 1.95) (11). Additionally, Lin et al. identified a positive link between SIRI levels and all-cause mortality among psoriasis patients (12). Therefore, we posited a potential inverse relationship between the LC9 score and the risk of psoriasis and examined the intermediary function of SIRI in this association. The objective was to enhance early intervention strategies and prevention of psoriasis through proactive.

## 2 Methods

### 2.1 Data source and population

NHANES is a continuous and representative comprehensive cross-sectional survey programme designed to assess the health and nutritional status of people in the U.S. NHANES has been approved by the Research Ethics Review Board of the U.S. National Center for Health Statistics. All participants in the NHANES surveys provided written informed consent, which was obtained by the NHANES study team as part of their data collection protocol (13).

**Figure 1:**
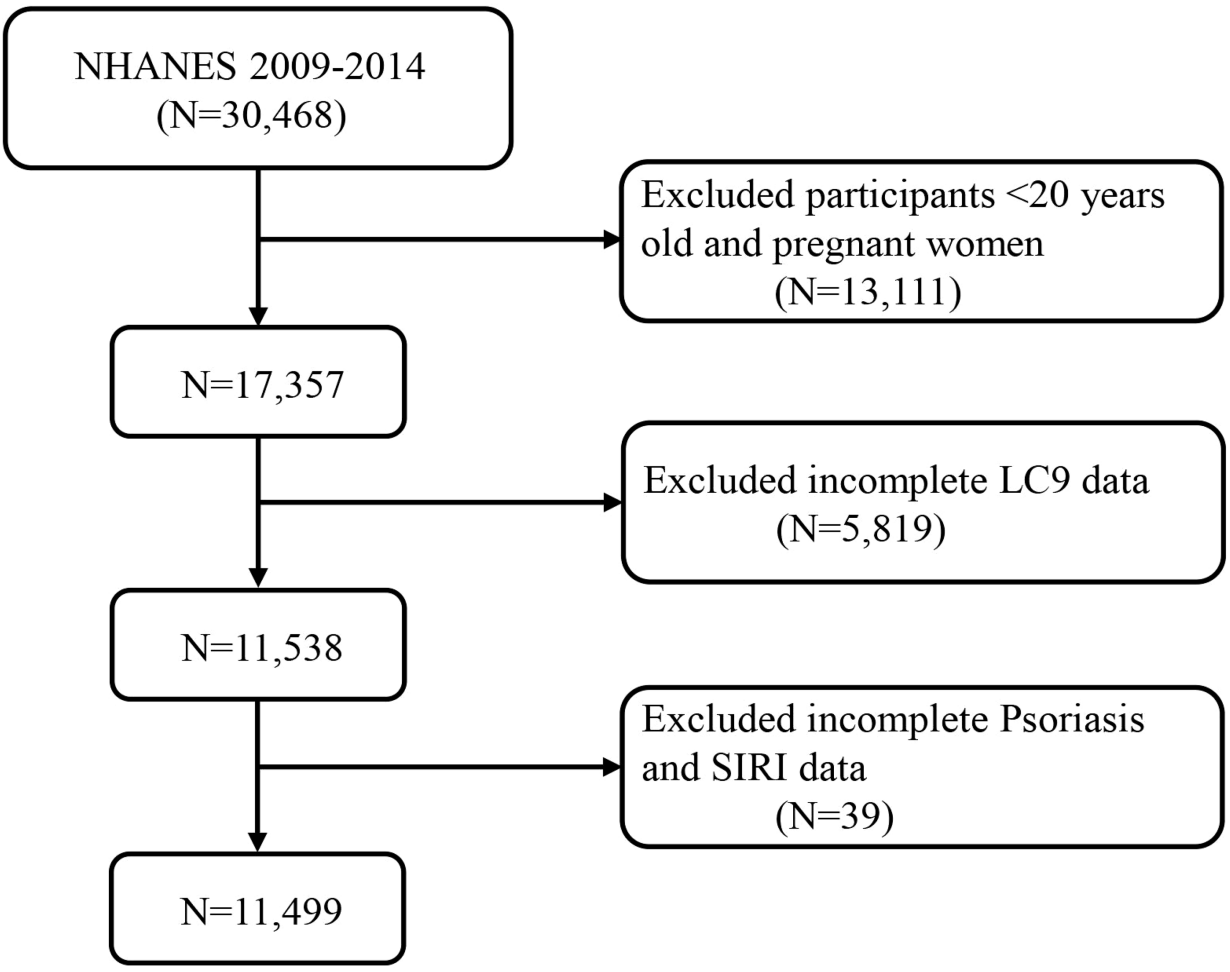
A flow diagram of eligible participant selection in the National Health and Nutrition Examination Survey. LC9, Life’s Crucial 9; SIRI, systemic inflammatory response index.

The study acquired data from 30,468 participants from the 2009-2014 cycle. The study screening removed individuals under 20 years old and pregnant women (n = 13111), missing LC9 data (n = 5819), and missing SIRI and psoriasis data (n = 39), leaving 11,499 subjects for inclusion in the analysis (**Figure 1**).

### 2.2 Calculation of the LC9 score

The LC9 consists of the following nine components: diet, physical activity, nicotine exposure, sleep health, BMI, blood lipids, blood glucose, blood pressure and psychological health (14). Each cardiovascular health (CVH) factor receives a normalized score between 0 and 100. The composite LC9 score is calculated as the mean of these normalized scores across the nine indicators. Nutritional metrics were evaluated based on the 2015 Healthy Eating Standards, while mental health evaluations derived scores from the Patient Health Questionnaire 9 (PHQ-9) (10). In brief, the specific calculations for each indicator refer to previous studies and are shown in the **Supplementary Materials** (**Supplementary Tables S1**, **S2**).

### 2.3 Definition of psoriasis and SIRI

Based on previous studies, the diagnostic criteria for psoriasis was assessed using NHANES self-reported questionnaires. Psoriasis is diagnosed by a specialized dermatologist who performs a careful morphological evaluation of the skin lesions following the pattern recognition method described by Raychaudhuri et al (15). NHANES survey gathered essential data by querying participants with the question, “Have you ever been told by a doctor or healthcare provider that you have psoriasis?” Those who answered “Yes” were categorized as psoriasis patients, and all others were excluded.

SIRI is a novel composite inflammation indicator based on routine blood tests that reflects an individual’s inflammatory state (16). Measured using an automated hematology analyzer, the formula for calculating SIRI is as follows: SIRI = monocyte count × neutrophil count/lymphocyte count. Values for cell counts are expressed as ×10^9^ cells/L.

### 2.4 Covariates

Based on previous studies (15, 17), covariates in this study included age, gender, ethnicity, marital status, educational attainment, PIR, hypertension, diabetes, and hyperlipidemia. Detailed information on these covariates is presented in **Supplementary Table S3**.

### 2.5 Statistical analysis

Sampling weights were used to ensure that the data were nationally representative, as recommended by the official NHANES website. This is calculated as follows: Weight = WTMEC2YR/3. Variables that followed a normal distribution were reported using the mean and standard deviation (SD), while those deviating from normality were described using the median and interquartile range. In terms of categorical data, these were presented as counts and percentages. To evaluate distinctions between individuals with and without psoriasis, the analysis employed the weighted Student’s t-test, Mann-Whitney U-test, and chi-square test, depending on the data’s distribution. In this research, weighted multivariate logistic regression analyzed the link between LC9 and psoriasis, while weighted linear regression explored the connection between LC9 and SIRI. The initial model, Model 1, did not incorporate adjustments for any confounding variables. Model 2 incorporated adjustments for demographic factors such as age, gender, ethnicity, marital status, educational attainment, and economic conditions. Model 3 expanded these adjustments to include medical conditions: hypertension, diabetes, and hyperlipidemia. The relationship between LC9 levels and psoriasis, including the role of potential mediators, was examined using restricted cubic spline (RCS) regression methods. To further dissect the direct influence of LC9 on the likelihood of psoriasis and the role of SIRI as an intermediary, analyses were conducted using the “mediation” package in the R statistical software. In addition, in order to investigate the impact of different populations and to reduce the effect of missing variables on the results, we further conducted sensitivity analyses including subgroup analyses and multiple interpolation. Missing values were interpolated using multiple interpolation via chained equations, thus generating five interpolated datasets based on the variables in the final statistical model. And we performed a WQS analysis to assess the combined and individual effects of the indicators in LC9 on psoriasis by calculating weighted linear indices and assigning corresponding weights.

## 3 Results

### 3.1 Baseline characteristics

A total of 11,499 participants aged 20 years and older were enrolled in this study, which included 345 individuals with psoriasis, a prevalence of 3.0%. The baseline characteristics of the participants according to whether they had psoriasis are shown in **Table 1**. Mean LC9 scores were 68.10 ± 1.04 in the psoriasis group and 71.08± 0.28 in the non-psoriasis group. The psoriasis group had a greater proportion of female, white-race participants compared to the non-psoriasis group. In addition, people with psoriasis were more likely to have higher levels of SIRI.

**Table 1:** Baseline characteristics of all participants were stratified by psoriasis, weighted.

Characteristic	Total	Non-Psoriasis	Psoriasis	P-value
No. of participants in the sample	11,499	11,154	345	-
LC9	70.98 (0.29)	71.08 (0.28)	68.10 (1.04)	0.003
LC9, Tertile				0.06
T1	33.57 (30.95,36.19)	33.37 (31.83,34.91)	39.68 (33.18,46.18)	
T2	33.39 (30.64,36.14)	33.37 (32.11,34.63)	33.78 (28.37,39.18)	
T3	33.04 (29.80,36.28)	33.25 (31.31,35.20)	26.54 (20.31,32.77)	
SIRI	1.25 (0.02)	1.24 (0.02)	1.43 (0.05)	0.002
SIRI, Tertile				0.002
T1	33.26 (30.67,35.85)	33.53 (31.68,35.39)	24.79 (18.46,31.12)	
T2	33.41 (30.56,36.25)	33.56 (32.47,34.65)	28.65 (21.00,36.29)	
T3	33.34 (30.20,36.48)	32.90 (31.23,34.58)	46.56 (39.47,53.66)	
Age				0.11
20-40	34.60 (32.06,37.14)	34.86 (32.76,36.97)	26.58 (18.78,34.39)	
41-60	37.67 (34.06,41.28)	37.52 (35.98,39.05)	42.47 (34.67,50.27)	
>60	27.73 (25.16,30.30)	27.62 (26.29,28.95)	30.95 (24.62,37.27)	
Sex				<0.001
Female	51.20 (47.48,54.92)	51.20 (50.32,52.08)	51.19 (45.43,56.96)	
Male	48.80 (45.11,52.49)	48.80 (47.92,49.68)	48.81 (43.04,54.57)	
Race				< 0.001
Non-Hispanic White	70.93 (62.48,79.38)	70.62 (66.81,74.44)	80.45 (75.33,85.58)	
Non-Hispanic Black	10.54 (9.07,12.01)	10.69 (8.84,12.54)	5.92 (3.57, 8.26)	
Mexican American	7.36 (5.66, 9.06)	7.47 (5.54,9.41)	3.90 (1.51,6.28)	
Other	11.17 (9.90,12.43)	11.21 (9.66,12.76)	9.73 (6.73,12.74)	
Marital status				0.57
no	36.76 (34.50,39.01)	36.71 (34.71,38.72)	38.43 (32.97,43.89)	
yes	63.21 (57.37,69.05)	63.29 (61.28,65.29)	61.57 (56.11,67.03)	
Education				0.72
Below high school	14.66 (12.82,16.51)	14.70 (12.98,16.41)	13.93 (9.17,18.69)	
High School or above	85.28 (78.55,92.00)	85.30 (83.59,87.02)	86.07 (81.31,90.83)	
PIR				0.68
Poor	20.26 (18.22,22.30)	21.47 (19.33,23.62)	22.51 (16.38,28.64)	
Not Poor	73.95 (67.30,80.60)	78.53 (76.38,80.67)	77.49 (71.36,83.62)	
Hypertension				0.004
no	60.91 (56.44,65.37)	61.23 (59.54,62.91)	51.04 (44.06,58.03)	
yes	39.09 (35.60,42.59)	38.77 (37.09,40.46)	48.96 (41.97,55.94)	
Diabetes				0.01
no	87.62 (81.14,94.10)	87.81 (86.98,88.64)	81.81 (76.47,87.15)	
yes	12.38 (11.27,13.49)	12.19 (11.36,13.02)	18.19 (12.85,23.53)	
Hyperlipidemia				0.11
no	27.96 (25.77,30.14)	28.14 (26.63,29.66)	22.14 (15.35,28.93)	
yes	72.04 (66.29,77.80)	71.86 (70.34,73.37)	77.86 (71.07,84.65)	

Mean (SD) for continuous variables: the P value was calculated by the weighted Students T-test.

Percentages (95%CI) for categorical variables: the P value was calculated by the weighted chi-square test.

LC9, Life’s Crucial 9; SIRI, systemic inflammatory response index; PIR, poverty income ratio.

### 3.2 Correlation between LC9 score and psoriasis

**Figure 2:**
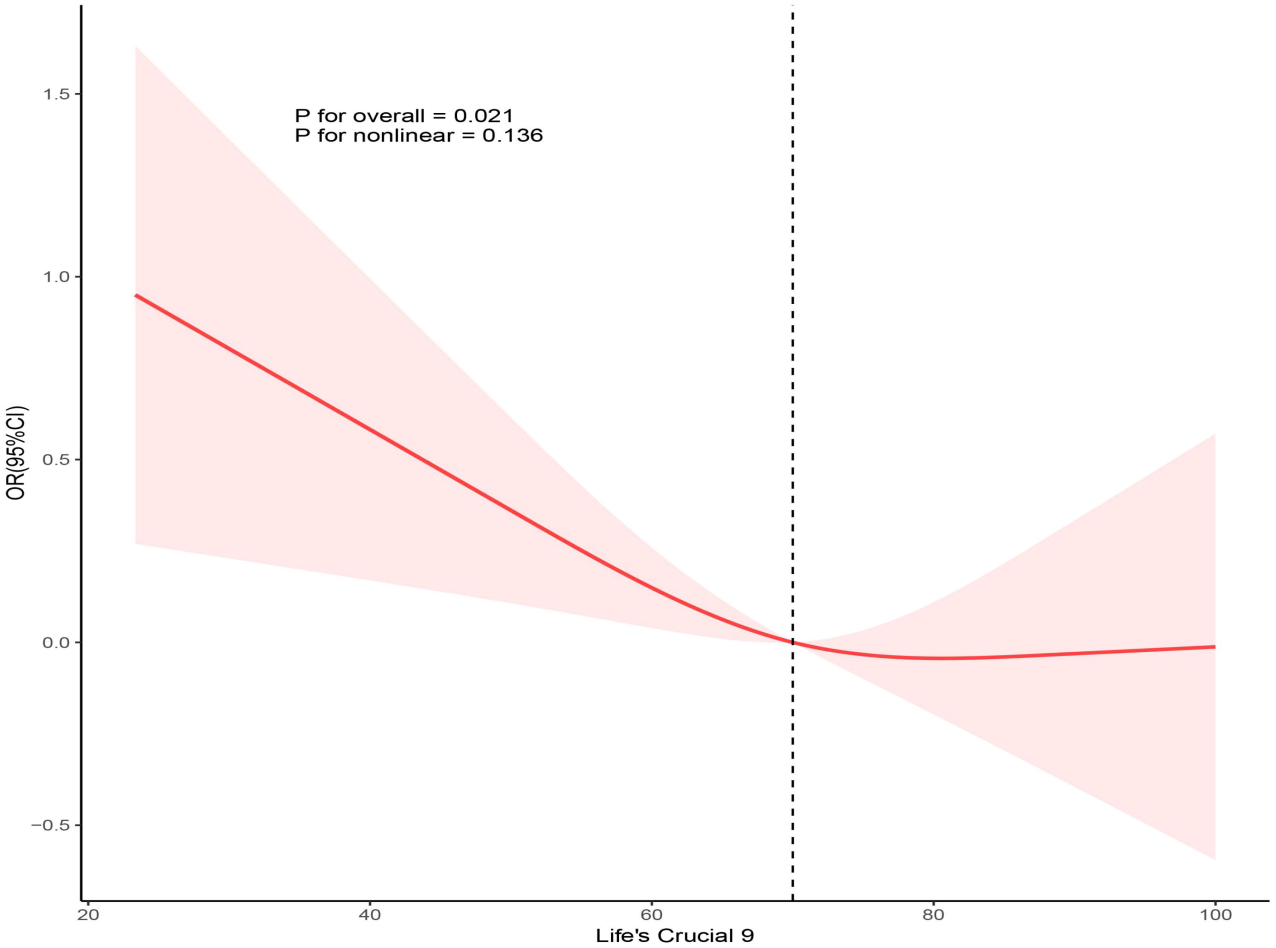
Dose-response relationships between LC9 and psoriasis. OR (solid lines) and 95% confidence levels (shaded areas) were adjusted for age, gender, education level, marital, PIR, race, hypertension, diabetes, and hyperlipidemia.

**Figure 3:**
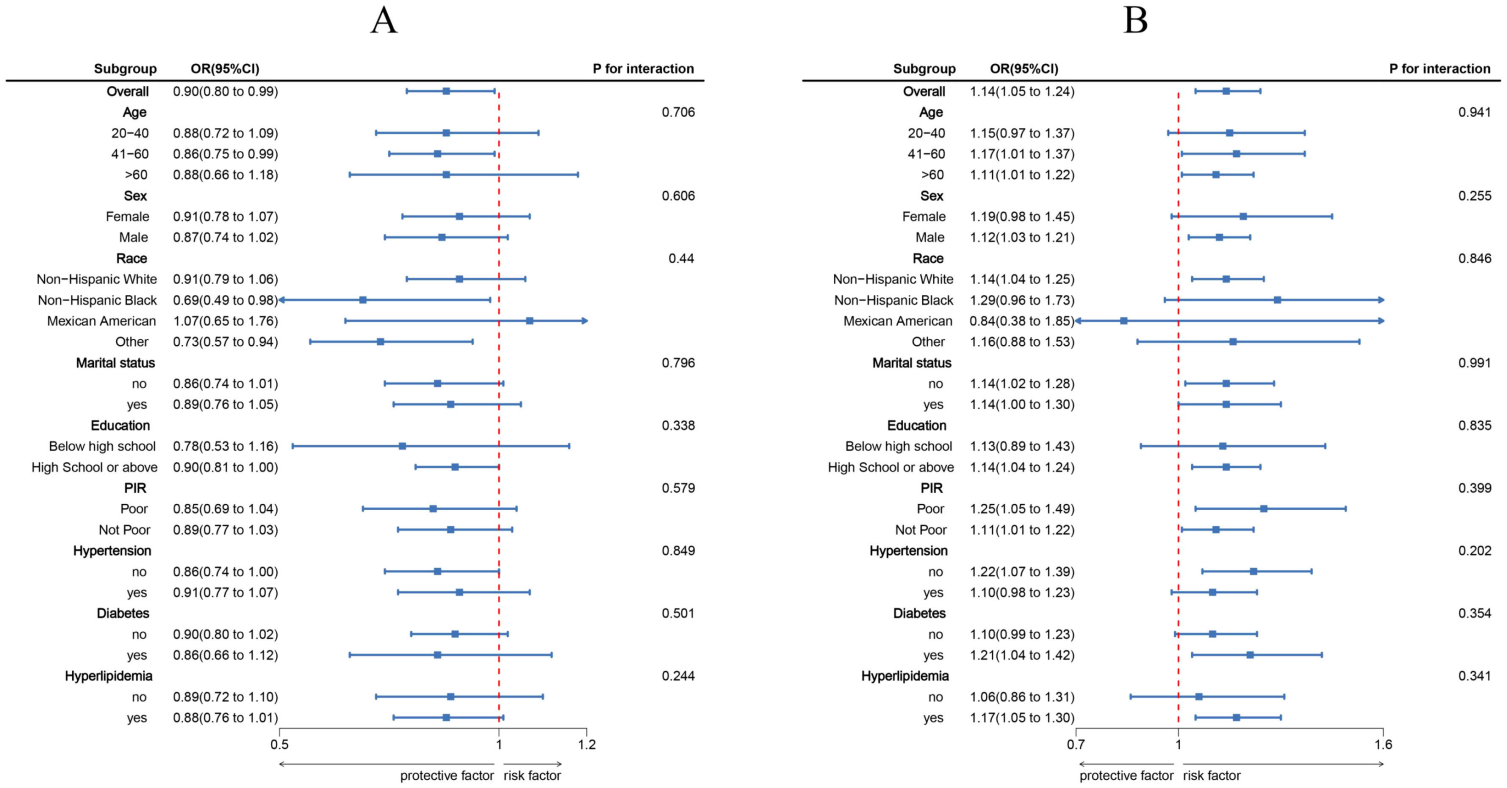
Subgroup analysis between LC9, SIRI, and psoriasis. **(A)** LC9 - Psoriasis; **(B)** SIRI - Psoriasis. ORs were calculated per 10-unit increase in LC9, and each standard deviation increased in SIRI. Analyses were adjusted for age, gender, education level, marital, PIR, race, hypertension, diabetes, and hyperlipidemia.

**Figure 4:**
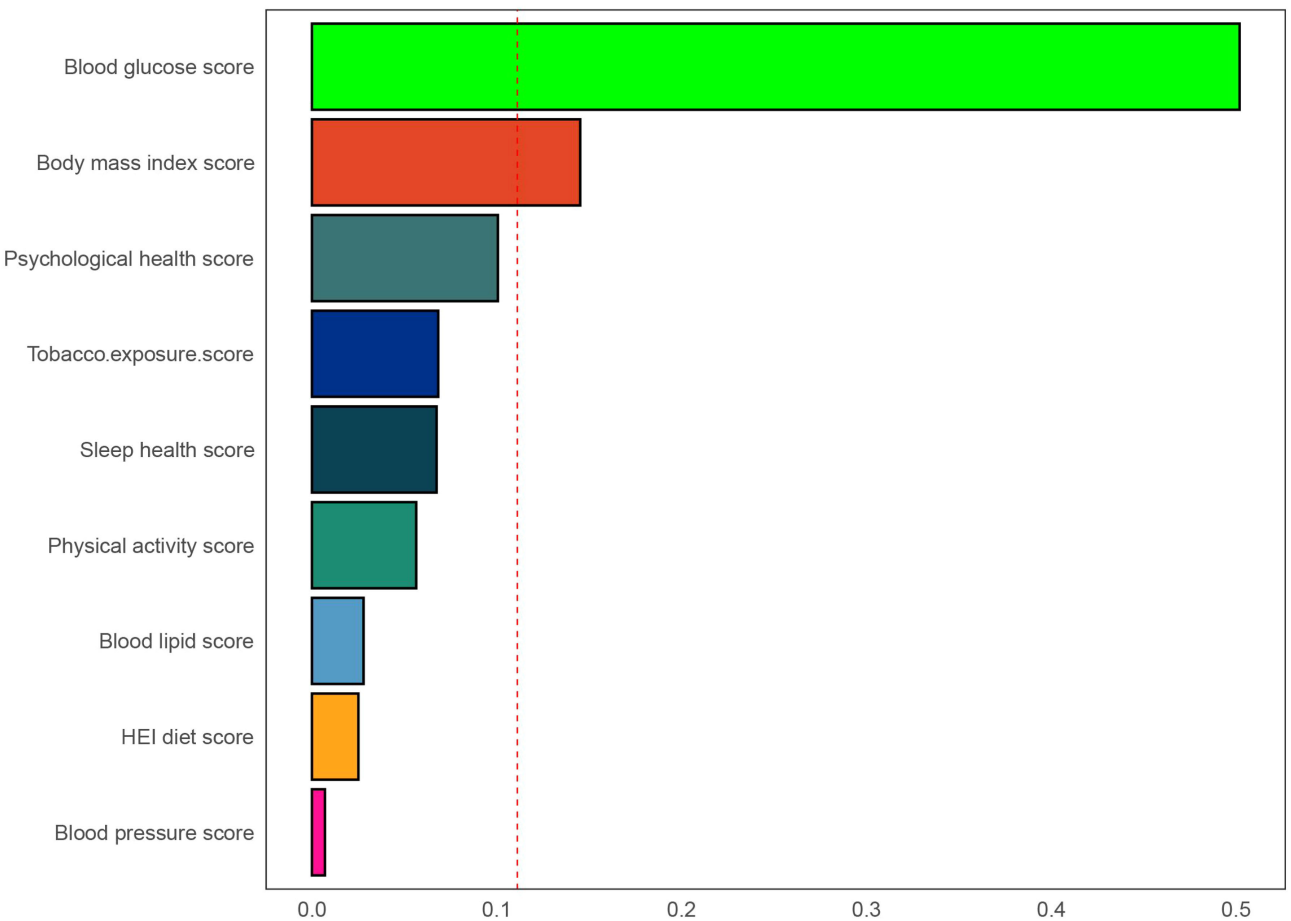
Weights represent the proportion of partial effect for each LC9 metric in the WQS regression. The model adjusted for age, gender, education level, marital, PIR, race, hypertension, diabetes, and hyperlipidemia.

Using three weighted logistic regression models, the results in **Table 2** show a correlation between LC9 scores and psoriasis in US adults. In fully adjusted model 3, a 10-point rise in LC9 score was associated with a 10% reduction in the risk of developing psoriasis (OR = 0.90, 95% CI: 0.80, 0.99). In addition, compared to the first tertile (T1), the odds of psoriasis were reduced by 24% (OR = 0.76, 95% CI: 0.58, 0.95) in Model 3 adjusted for all covariates. In addition, after multiple imputation, the associations between LC9 scores and psoriasis (OR = 0.90, 95% CI = 0.85,0.94) and remained significant in the fully adjusted model. Analyses across Models 1 and 2 showed consistent outcomes. **Figure 2** presents RCS analysis results, revealing a notable inverse relationship between LC9 scores and psoriasis risk. Additionally, to ensure the robustness of the LC9-psoriasis link across varying demographics, subgroup examinations were conducted using the framework of Model 3. The results are shown in **Figure 3**, and we did not find any significant interaction among the subgroups. In addition, **Figure 4** shows that all intra-LC9 metrics were negatively correlated with psoriasis, with glycemic metrics identified as the most influential factor influencing the development of psoriasis.

**Table 2:** Association between LC9, SIRI, and Psoriasis, NHANES 2009–2014.

Characteristics	Model 1 [OR (95% CI)]	P-value	Model 2 [OR (95% CI)]	P-value	Model 3 [OR (95% CI)]	P-value
LC9 - Psoriasis						
Continuous (per 10 scores)	0.86 (0.78,0.95)	0.003	0.86 (0.77,0.95)	0.007	0.90 (0.80,0.99)	0.011
Tertile						
T1	1 (ref.)		1 (ref.)		1 (ref.)	
T2	0.85 (0.64,1.13)	0.260	0.88 (0.67,1.17)	0.379	0.97 (0.72,1.30)	0.663
T3	0.67 (0.47,0.95)	0.027	0.64 (0.43,0.97)	0.037	0.76 (0.58,0.95)	0.025
* P for trend*	0.024		0.030		0.036	
SIRI - Psoriasis						
Continuous	1.17 (1.09,1.26)	<0.001	1.16 (1.06,1.25)	0.001	1.14 (1.05,1.24)	0.003
Tertile						
T1	1 (ref.)		1 (ref.)		1 (ref.)	
T2	1.15 (0.72,1.84)	0.540	1.15 (0.68,1.96)	0.600	1.14 (0.66,1.94)	0.630
T3	1.91 (1.35,2.71)	<0.001	1.86 (1.21,2.84)	0.010	1.77 (1.15,2.72)	0.010
* P for trend*	<0.001		0.003		0.010	

Model 1: no covariates were adjusted.

Model 2: age, gender, education level, marital, PIR, and race were adjusted.

Model 3: age, gender, education level, marital, PIR, race, hypertension, diabetes, and hyperlipidemia were adjusted.

LC9, Life’s Crucial 9; SIRI, systemic inflammatory response index; PIR, poverty income ratio; OR, odds ratio; CI, confidence interval.

### 3.3 SIRI and risk of psoriasis

**Table 2** illustrates the link between SIRI and psoriasis, with univariate logistic regression indicating an inverse association (OR: 1.17, 95% CI: 1.09, 1.26). This relationship was consistently significant in all three models. Even after controlling for all covariates, the negative association between SIRI and psoriasis remained statistically significant (OR = 1.14, 95% CI: 1.05, 1.24). After stratification into tertiles of SIRI, individuals in the highest tertile (T3) were 0.77 times more likely to develop psoriasis compared to the lowest tertile (T1) (OR: 1.77, 95% CI: 1.15, 2.25). CI: 1.15, 2.72). In addition, after multiple imputation, the associations between DI-GM and constipation (crude model: OR = 0.81, 95% CI = 0.76, 0.86; adjusted model: OR = 0.83, 95% CI = 0.77, 0.89) and DI-GM ≥ 6 (adjusted model: OR = 0.5, 95% CI = 0.37, 0.69) remained significant (**Supplementary Table S4**). Adjusting for multivariate RCS regression revealed a linear association between SIRI and psoriasis (**Supplementary Figure S1**).

### 3.4 Association of LC9 and SIRI

**Table 3** shows that further exploration indicated an association between LC9 and SIRI. We observed that LC9 was negatively associated with SIRI (β = -0.08,95% CI: -0.09, -0.07, p < 0.001). In addition, after multiple imputation, the associations between LC9 and SIRI (β = -0.07,95% CI: -0.08, -0.06, p < 0.001) remained significant (**Supplementary Table S5**).

**Table 3:** Multivariate linear regression of LC9 and SIRI.

	β	95%CI	P-value
LC9 - SIRI	-0.08	(-0.09, -0.07)	<0.001

Adjusted for age, gender, education level, marital, PIR, race, hypertension, diabetes, and hyperlipidemia.

### 3.5 Mediation effect

**Figure 5:**
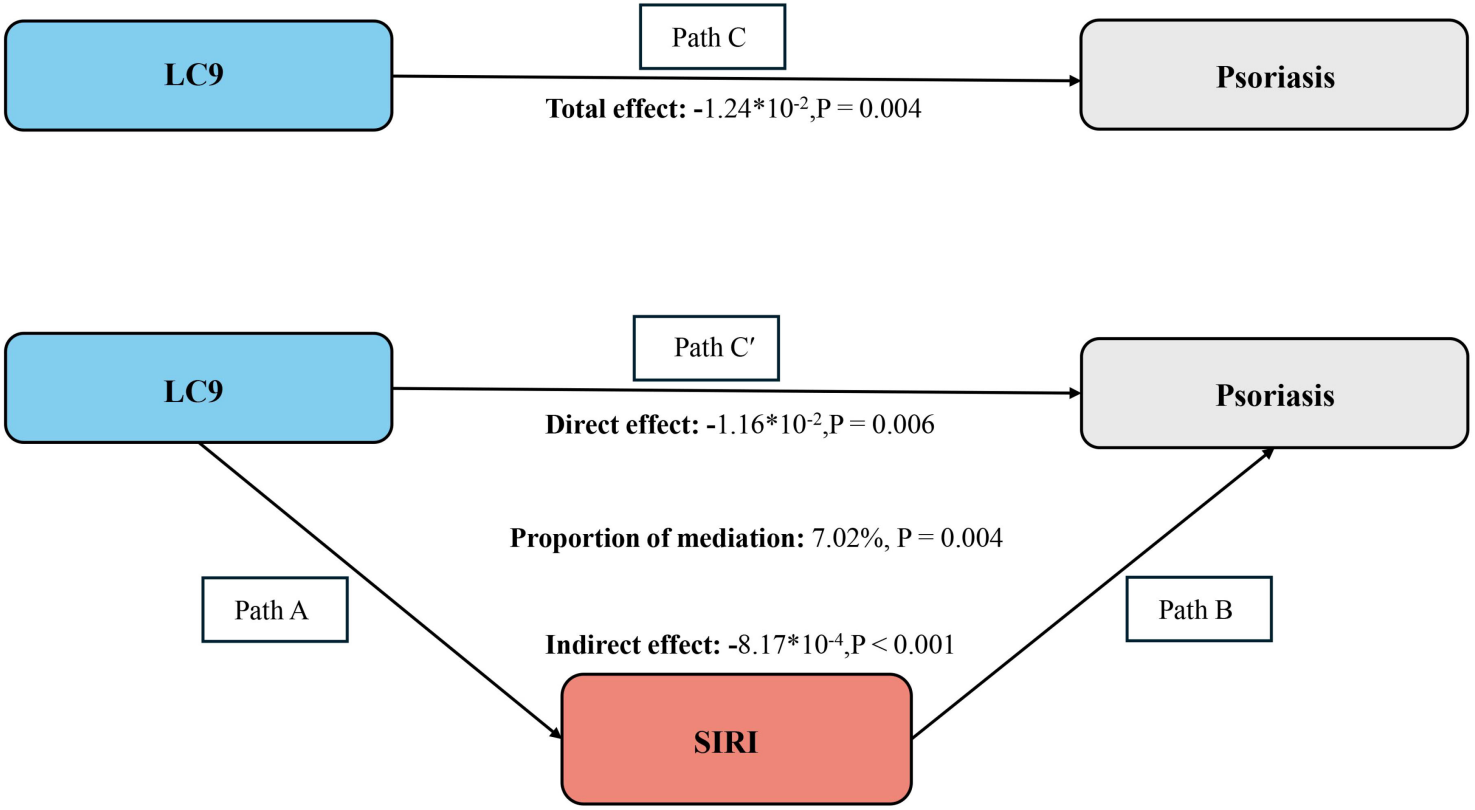
Schematic diagram of the mediation effect analysis. Path C indicates the total effect; path C′ indicates the direct effect. The indirect effect is estimated as the multiplication of paths A and B (path A*B). The mediated proportion is calculated as indirect effect/(indirect effect + direct effect) × 100%. LC9, Life’s Crucial 9; SIRI, systemic inflammatory response index. Analyses were adjusted for age, gender, education level, marital, PIR, race, hypertension, diabetes, and hyperlipidemia.

After accounting for all covariates, our analysis extended to examine SIRI’s intermediary function in the link between LC9 and psoriasis. The findings from this exploration are depicted in **Figure 5**. We observed a total effect of LC9 on psoriasis of -1.24*10^-2^ (p = 0.004). The results showed that SIRI mediated 7.02% of the complex association between LC9 and psoriasis.

## 4 Discussion

Utilizing data from the NHANES 2009-2014 dataset, which included 11,499 U.S. adults, this study investigated the link between LC9 scores and psoriasis risk. Additionally, the analysis focused on SIRI’s mediating influence in this association. Findings indicated a notably inverse relationship between LC9 scores and the probability of psoriasis onset. Furthermore, SIRI played an important mediating role between LC9 and psoriasis. Therefore, the findings suggest that early monitoring and management of LC9 scores may help identify individuals at high risk for psoriasis. Individuals may also be able to improve inflammation levels in the body through lifestyle modifications, ultimately reducing psoriasis risk. In addition, the results of the WQS regression analysis further emphasize the critical role of glycemic health in the prevention of psoriasis.

Psoriasis is a common chronic autoimmune inflammatory disease with a complex pathogenesis involving environmental, immunological and genetic factors (18, 19). The SIRI is an inflammatory biomarker used to comprehensively assess the collective systemic inflammatory state and immune homeostasis (20). In recent years, SIRI has been demonstrated to be useful in predicting diseases such as colorectal cancer, stroke, and coronary heart disease (21–23). It has been shown that SIRI levels are positively correlated with psoriasis risk in the US population (11). Our study also reaffirms the role of SIRI in psoriasis.

Psoriasis used to be viewed as a skin disease only, but recent studies have revealed strong links to systems and diseases such as the cardiovascular system, the metabolic syndrome, and the psychiatric system, etc (24). The results of a large prospective cohort study by Wang et al. suggest that poor mental health is associated with an increased risk of cardiovascular disease (25). Multiple mental health factors, including depression, anxiety, and chronic stress, were independently associated with CVD risk. Recently, Allison et al. proposed the inclusion of mental health in the Life’s Essential factor, updating the metric to Life’s Crucial 9 (14). Studies have demonstrated the predictive value of the LC9 for new cardiovascular mortality. All of the above emphasize the important role of mental health factors in CVD disease.

Our findings can be explained by possible mechanisms. Firstly, psoriasis and cardiovascular disease (CVD) share common inflammatory pathways. Chronic systemic inflammation in psoriasis is mediated by cytokines such as tumor necrosis factor (TNF), interleukin (IL-17, and IL-23), which are also involved in atherosclerosis and other cardiovascular diseases (1). Lifestyle factors significantly influence the development of psoriasis. A large-scale cohort study of 487,835 Japanese adults demonstrated that both physical inactivity (defined as <1 hour of moderate-to-vigorous exercise weekly) and higher BMI independently increased psoriasis risk, with hazard ratios of 1.13 (95% CI: 1.05-1.22) and 1.09 (95% CI: 1.05-1.14), respectively (26). Some studies have shown that serum levels of IL-17 are elevated in patients with metabolic syndrome compared to normal subjects (27). Liu et al. found a positive correlation between the lipid accumulation product (LAP) index and the prevalence of psoriasis in young men. In addition, some studies have suggested that psychological stress can cause or exacerbate psoriasis (28, 29). Acute stress can induce lymphocyte aggregation, and long-term chronic stress suppresses the body’s immune function (30).

Maintaining optimal cardiovascular health through self-management can reduce the risk of psoriasis. Engaging in regular physical activity and ensuring sufficient sleep can mitigate the risk of psoriasis by reducing body weight, enhancing antioxidant enzyme activity, and alleviating psychological stress (31–33). Smoking induces the activation of keratinocytes and innate immune cells, producing inflammatory factors such as tumour necrosis factor-α, IL-6 and IL-2 (34, 35). A recent Korean real-world study confirms that quitting smoking reduces the risk of psoriasis (36). Additionally, a diet rich in dietary fiber, antioxidants, and polyphenols is associated with a decreased risk of psoriasis due to its protective properties (37). Metabolic syndrome leads to the release of multiple inflammatory factors and adipokines that promote oxidative stress and lead to endothelial dysfunction (19, 38, 39). Studies have shown that patients with a lower body mass index and better control of lipids and blood pressure (better CVH) have a lower incidence and severity of psoriasis (40).

A meta-analysis showed that psoriasis patients randomized to glucose-lowering medications had significantly lower Psoriasis Area and Severity Index (PASI) scores and were more likely to achieve a PASI of 75 (41). Hyperglycemia, as a central driver of the diabetic process, may impair vascular endothelial function, and combined with insulin resistance plays a potential role in the development of psoriasis, however further studies are still needed to confirm this (42).

Previous studies have shown significant aggregation of neutrophils and platelets in the blood of psoriasis patients (43).Activated neutrophils can promote the secretion of various cytokines and chemokines, such as TNF and IL-17, by inducing oxidative stress, degranulating and releasing various compounds such as myeloperoxidase (44). Dendritic cells and macrophages in the dermis of psoriasis produce IL-23, which induces the activation of Th17 cells and γδ T cells and the release of inflammatory cytokines, such as IL-17A, IL-17F, IL-22, IL-6 and tumor necrosis factor-α (TNF-α). The cytokines act on keratinocytes, leading to epidermal hyperplasia, stratum corneum hypertrophy and hyperkeratosis, which are typical pathological changes in psoriasis (45). In recent years, it has been widely recognized that psoriasis is an immune-mediated systemic disease involving cardiovascular comorbidities, metabolic disorders and atherosclerotic disease (46). The results of previous studies by the group have shown that the non-HDL cholesterol to HDL cholesterol ratio (NHHR) is significantly associated with the risk of psoriasis (47). In addition, the latest findings also suggest that NLRP3 inflammatory vesicles are closely associated (48). SIRI, as a comprehensive inflammatory index, reflects the state of systemic inflammation, but its calculation is based on neutrophil, monocyte, and lymphocyte counts, which do not directly reflect the dynamics of inflammation or the functional status of specific cell subpopulations. Thus, although SIRI showed some correlation in psoriasis, its limited explanatory power as a mediating variable may not be sufficient to fully reveal the complex association between LC9 and psoriasis.

The strengths of this study are as follows: to our knowledge, this is the first to explore the association between LC9 and psoriasis and the mediating role of SIRI in large U.S. population dataset. the LC9 across a variety of lifestyle and health indicators allows for a more systematic and comprehensive assessment of cardiovascular health. We used weighted data to reveal a negative association between LC9 score and risk of psoriasis in the US adult population. This could help policy makers develop intervention strategies to improve health behaviors and increase LC9 scores in the population, thereby reducing the disease burden of psoriasis. We found that SIRI has a mediating role between LC9 and psoriasis. Thus, SIRI can help provide a simple method to identify high-risk populations and target healthy lifestyle guidance to individuals.

However, there are some limitations to this study of ours. Firstly, although we have controlled for multiple confounders, we cannot completely exclude confounders that may affect psoriasis, such as genetic background, environmental exposure, and drug use. Second, the diagnosis of psoriasis and outcomes such as diet and depression relied on questionnaire moderation, which may have been affected by recall bias. Additionally, the NHANES database lacks information on psoriasis severity scores, limiting our ability to assess the potential modifying effect of psoriasis activity on the LC9-inflammation association. And the generalizability of the results needs to be validated in combination with clinical cohorts in the future. In addition, SIRI cannot suggest the dynamic changes of inflammation and reflect the subpopulation and functional status of cell types. Therefore, in clinical applications, SIRI usually needs to be combined with other inflammatory markers or clinical symptoms to comprehensively assess the inflammatory status. Most importantly, we cannot ascertain the causal relationship between LC9 and psoriasis, nor the mechanism by which SIRI is involved. Therefore, experimental studies are needed in the future to elucidate these aspects.

## 5 Conclusions

In conclusion, lower LC9 scores increase the risk of having psoriasis, in which SIRI plays a mediating role. This emphasizes the potential role of early lifestyle and mental health management in preventing and delaying the onset of psoriasis. It contributes to better public health decision-making to reduce the disease burden of psoriasis.

## Data Availability

Publicly available datasets were analyzed in this study. The complete dataset is accessible through the official NHANES website at https://www.cdc.gov/nchs/nhanes/index.htm.
